# Lack of Knowledge About Hepatitis C Infection Rates Among Patients With Inherited Coagulation Disorders in Countries Under the Eastern Mediterranean Region Office of WHO (EMRO): A Meta-Analysis

**DOI:** 10.5812/hepatmon.844

**Published:** 2012-04-30

**Authors:** Seyed Moayed Alavian, Seyed Hossein Aalaei-Andabili

**Affiliations:** 1 Baqiyatallah University of Medical Sciences, Baqiyatallah Research Center for Gastroenterology and Liver Disease (BRCGL), Tehran, IR Iran

**Keywords:** Meta-analysis, Hemophilia, Epidemiology, Blood Coagulation Disorders, Iran

## Abstract

**Context:**

Hepatitis C virus (HCV) infection is a public health problem. HCV alone is responsible for 90% cases of acute hepatitis among multiply transfused patients who are at risk of cirrhosis and hepatocellular carcinoma (HCC).

**Objectives:**

To provide a clear picture of available data, we performed a systematic review of the epidemiological characteristics of HCV infection among patients with inherited coagulation disorders in the countries under the Eastern Mediterranean Region Office (EMRO).

**Evidence Acquisition:**

Meta-analysis was carried out on the basis of results of electronic and manual search. This analysis included studies in English, French, and Persian that met with the following criteria: (1) appropriate study design: cross-sectional, case-control, and cohort; (2) studies reporting HCV prevalence according to enzyme immune assay; (3) studies in which the sample population was enrolled from EMRO countries. According to the results of the heterogeneity tests, we used fixed-effect/random-effect model for our meta-analysis, with the “Metan” command.

**Results:**

We included 30 studies, most of which were from Iran. The mean age of the subjects ranged from 13 to 27.1 years. The majority of the patients were male (range, 81% to 100%). The pooled estimate of HCV infection among patients with inherited coagulation disorders was 48.07% (95% confidence interval [CI], range: 27.39–55.68) in Iran, 36.03% (95% CI, range: 4.466–67.598) in Pakistan, and 48.27% (95% CI, range: 36.12–60.43) in all the EMRO countries taken together.

**Conclusions:**

In Iran and other EMRO countries, the HCV infection rate among patients with inherited coagulation disorders is high. Our study shows that there is a lack of knowledge about infections in such patients in most of the EMRO countries. It is the responsibility of health-policy makers to address this knowledge gap and provide safe and adequate treatment for patients in high-risk groups.

## 1. Context

Hepatitis C virus (HCV) infection is a common global public health problem [1][2]. Approximately 200 million individuals are infected with HCV worldwide [3]. Although the prevalence of HCV infections has reduced in developed countries because of effective prevention plans [4], it is still high in developing countries [5]. HCV is considered as the main cause of liver diseases in both developed and developing countries and contributes to the increasing risk of liver failure and hepatocellular carcinoma (HCC) [3][4][6][7]. In addition, HCV is responsible for 20% of all acute hepatitis cases, 70% of all chronic hepatitis cases, 40% of all liver cirrhosis cases, 60% of HCCs, and 30% of infections in liver transplants in Europe [8][9]. Moreover, most of the recently admitted HCC patients had viral hepatitis C [10]. Hemophilia is the most common inherited congenital bleeding disorder and it is estimated to affect between 1 to 5 individuals per 50,000 male population [11]. Hepatitis C is considered responsible for post-transfusion hepatitis and most of the observed liver diseases in treated hemophilia patients [12]. All multiply transfused patients who received clotting-factor concentrates before 1985 and/or blood transfusions before 1992 are infected with Hepatitis C, and almost all of them have tested positive for the HCV antibody. The prevalence of hepatitis C among hemophilic patients varies from 24% to 95% across countries [9][13][14][15][16][17][18], and it is reported to range from 13.3% to 80.5% in Iran [19][20][21]. Hepatitis C virus alone is responsible for 90% of acute hepatitis infections in these multiply transfused patients, and 60% to 80% of these infections progress to chronic hepatitis [22].

## 2. Objectives

In the USA and European countries, prominent changes have been observed in the epidemiology of hepatitis C [23][24]. We designed a literature review to determine epidemiological changes in the countries included in the Eastern Mediterranean Region Office of WHO (EMRO). For a clear and comprehensive presentation of the available data, we conducted a systematic review of the epidemiological characteristics of hepatitis C to determine epidemiological changes in HCV infection among patients with inherited coagulation bleeding disorders in EMRO countries.

## 3. Evidence Acquisition

### 3.1. Search Strategy

We performed an electronic search of the available content and a manual search of specialty journals and congress books to find all pertinent literature. We thought manual search was necessary because not all published data are available online. We began with an electronic search on the MEDLINE database Pubmed, Scopus, Ovid, Embase, and ISI. Then, we performed searches on Google Scholar and continued until the 200th link without finding any related article. We also performed Google searches to find gray literature. For Iranian articles, Persian databases such as IranMedex, Scientific Information Database, and magIran were searched as we did it for all Iranian abstract books of the Iranian congresses. In addition, we searched all the related references in the articles found, and contacted some corresponding authors by e-mail to obtain the full text of their article. We continued our search until it reached a saturation level and checked our search sensitivity by counting the duplications. We used terms such as “hemophilia” or “haemophilia” or “congenital inherited bleeding disease” and “hepatitis C” or “chronic hepatitis C” or “HCV,” the names of countries (Afghanistan, Bahrain, Djibouti, Egypt, Iran, Iraq, Jordan, Kuwait, Lebanon, Libya, Morocco, Oman, Pakistan, Palestine, Qatar, Saudi Arabia, Somalia, Sudan, Syria, Tunisia, United Arab Emirates, and Yemen), and Persian words as key words for Iran-specific searches. There were no limitations in our search. We tried to include all published studies in the English, French, and Persian languages till the end of 2011.

### 3.2. Data Extraction and Quality Assessment

Data extraction was performed independently by 1 investigator (SH. AA), and critical appraisal for quality assessment of the articles was performed with JAMA,form by who were not involved in the search (SM A). We coordinated meetings before the critical appraisal and investigators have been justified about questions. After critical appraisal, selected articles were checked by both the authors.

### 3.3. Inclusion and Exclusion Criteria

Published/unpublished studies in English, French, and Persian were included if they met the following criteria: [1] studies with an appropriate design: cross-sectional (C-S), case-control (C-C), and cohort; [2] studies that reported HCV prevalence as determined on the basis of the results of enzyme immune assays 3) studies in which the sample population was enrolled from EMRO countries. The exclusion criteria were as follows: [1] studies with probable errors and confusing data 2) studies in which only PCR findings were reported . Authors’ names or journal names did not influence our decision in excluding or including the articles. We extracted the first author’s name, the year of publication, name of the country, type of study, number of patients included in the study, patients’ characteristics such as mean age, male to female ratio, prevalence, and subgroup prevalence from the selected studies, if full texts of the articles were available.

### 3.4. Statistical Analyses

e analyzed the extracted data to estimate the pooled prevalence of HCV infection and its 95% confidence interval (CI) in Iran, Pakistan, Tunisia, and Iraq, and in all the EMRO countries taken together. Statistical heterogeneity of the results was evaluated using heterogenicity tests, such as Q-squared test, I-squared test, and Tau-squared statistics. For Q-squared test, a P value of <0.1 was considered as significant. I-squared value lies between 0% to 100% and the heterogeneity increases with increasing I-squared value. Since not many articles were included, especially in our subgroups, we thought that the Tausquared test was more suitable for our study because it is not influenced by the number of included studies [25]. Depending on the results of the heterogeneity tests, we used a fixed-effect/random-effect model for our meta-analysis with the “Metan” command. The analysis was performed using Stata 11.

## 4. Results

### 4.1. Search Result and Study Selection

Our findings showed that there are not enough data with respect to HCV infections among inherited coagulation disorders from the developing countries of EMRO. Most of the EMRO countries did not report the number of hemophilic patients, and it varies from 1 per 100,000 male population in Saudi Arabia and Pakistan to 15.8 per 100,000 male population in Qatar (Table 1) [26]. In addition, the available data are not up to date, and in most of the studies sample size is small and the quality of methodology poor. Through our searches of titles and abstracts, we found 39 potentially relevant studies that had evaluated anti-HCV seroprevalence in patients with inherited coagulation disorders in EMRO countries. All the studies were carefully evaluated to avoid including duplicates or low-quality papers. Two letters to editors [27][28], and 1 review article were excluded [29]. One study was excluded because of low sample size. [30] Another study was excluded because the full text was not available and there was not enough information in the abstract. Furthermore, we did not receive any response from the authors of the article after 1 month of our e-mail request for the full text [31]. In addition, we excluded an article because of its selection bias and overestimation as it was reported previously [32], although there was enough data and an acceptable sample size [33]. Three other studies were excluded because only PCR was performed to test for HCV infection [11][34][35]. Finally, 30 studies were selected for the analysis [19][20][21][36]-[62]. It was noteworthy that no data was available from Egypt although this country has the highest number of hemophilia patients (4141) in the eastern Meditarranean region [27]. In addition, we could not find any relevant study on HCV prevalence in patients with bleeding disorder from Bahrain, Kuwait, Jordan, Lebanon, Libya, Oman, Qatar, Emirates, Yemen, Sudan, Djibouti, Syria, Morocco, Somali, and Afghanistan. Eventually, we found 21 studies from Iran involving 3171 of a total of 3463 hemophilic patients, which accounted for more than 90% of all hemophilic patients in this country; 4 studies involving 841 subjects from Pakistan; 2 studies with 165 patients from Tunisia; 2 studies involving 290 patients from Iraq; and only 1 study from Saudi Arabia. The mean age of the subjects ranged from 13 to 27.1 years. The majority of the study population was male (range, 81% to 100%). Among the selected studies, 25 of them were C-S and the other 5 studies were C-C (Table 2).

**Table 1:** Mean Hemophilia Prevalence (per 100000 male population) Determined From the Reported Number of Hemophilic Patients in EMRO Countries in the Period 1998–2006 Divided by Its Male Population in the Corresponding Period

	**Iran**	**Iraq**	**Egypt**	**Jordan**	**Lebanon**	**Morocco**	**Pakistan**	**Palestine**	**Qatar**	**Saudi-Arabia**	**Sudan**	**Tunisia**
Mean	9.4	3.6	8.7	6.4	2.9	1.9	1.0	6.4	15.8	1.0	1.6	4.2
SD a	1.2	0.0	1.2	0.7	1.4	NR	0.5	1.9	NR	0.0	0.2	1.1

^a^ Abbreviation: SD; standard deviation

**Table 2:** Characteristics of Studies and Patients in EMRO Countries

	**Author**	**Date**	**Design**	**Sample Size**	**ELISA**	**Mean Age**	**Male, %**	**Prevalence, %**	**95% CI**
Iran									
	Samar G	1996	C-S a	102	2nd	18.55	95	77.50	69.40–85.60
	Ebrahim-Poor S	1997	C-C a	103	2nd	NR a	NR	76.7	68.54–84.86
	Hashemiyeh M	1997	C-S	44	NR	NR	NR	81.8	70.39–93.20
	Khamisipour GR	1999	C-S	31	NR	16	NR	41.9	24.53–59.27
	Alavian SM	2001	C-S	176	2nd	20.65	86	60.20	52.97–67.43
	Karimi M	2001	C-C	300	2nd	NR	NR	15.65	11.54–19.76
	Karimi M	2002	C-S	310	2nd	17	90	15	11.03–18.97
	Mansour-Ghanaei F	2002	C-S	101	2nd	19.7	99	71.30	62.48–80.12
	Zahedi MJ	2004	C-S	97	NR	21.8	87	44.30	34.41–54.18
	Ziaee M	2004	C-S	80	2nd	23	94	55	40.09–65.90
	Sharifi-Mood B	2006	C-S	74	NR	13	84	32.00	21.37–42.63
	Mohammad-Alizadeh AH	2006	C-S	66	3rd	24.6	83	59.10	47.23–70.96
	Torabi SE	2006	C-S	162	NR	18.5	89	51.20	41.98–60.41
	Javadzadeh-Shahshahani H	2006	C-S	74	3rd	22.5	93	48.60	37.21–59.98
	Sharifi-Mood B	2007	C-S	81	2nd	NR	NR	29.60	19.66–39.54
	Samimi-Rad K	2007	C-S	76	3rd	20.1	87	43.40	32.26–54.54
	Mojtabavi-Naini M	2007	C-S	553	2nd	23.4	84	22.60	19.11–26.09
	Mahdaviani FS	2008	C-S	68	3rd	20.3	85	36.7	25.24–48.15
	Mobini GR	2009	C-S	77	3rd	21.9	95	53.20	42.05–64.32
	Hedayat B	2009	C-S	30	3rd	NR	NR	13.30	1.15–25.45
	Kalantari H	2011	C-S	615	3rd	27.1	83	80.50	77.31–83.63
Iraq									
	Al-Kubaisy WA	2006	C-S	47	3rd	NR	NR	66.00	52.46–79.54
	Abdul-Karim ET	2011	C-S	243	NR	14.3	NR	40.30	34.13–46.47
Pakistan									
	Raihan S	2010	C-C	408	3rd	17	81	1.40	0.26–2.54
	Borhany M	2011	C-C	173	3rd	NR	100	51.4	43.95–58.85
	Naumaan M	2006	C-S	100	2nd & 3rd	NR	NR	56.00	46.27–65.73
	Asif N	2009	C-S	161	NR	NR	NR	36.00	28.59–43.41
Saudi Arabia									
	Bahakim H	1991	C-C	28	NR	NR	NR	78.60	63.41–93.79
Tunisia									
	Langar. H	2005	C-S	70	NR	19	100	55.70	44.06–67.34
	Djebbi A	2008	C-S	95	4th	19	100	55.70	45.71–65.69

^a^ Abbreviations: C-S, cross-sectional; C-C, case-control; NR, non-Reported; CI, confidence interval

### 4.2. HCV Infection Among Iranian Hemophilic Patients

**Figure 1:**
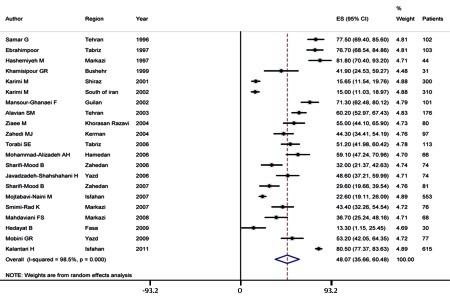
Summary of HCV Infection Rate Estimates in Hemophilic Patients in Iran Figure

**Figure 2:**
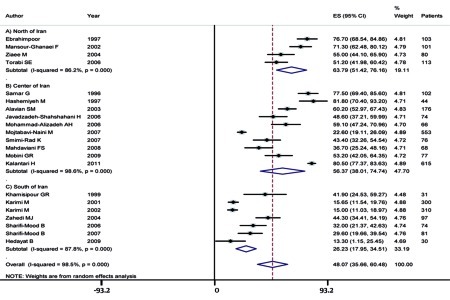
HCV Infection Rate Among Hemophilic Patients According to 3 Main Regions of Iran

The seroprevalence of HCV infection among hemophilic patients in Iran varies from 13.3% to 80.5% in point estimation, and the pooled estimation according to the random-effect model was 48.07% (95% CI, range: 35.66–60.48) with a P value of < 0.001 as determined by the Q-squared test, I2 = 98.6%, and Tau-square value = 816.8 (Figure 1). Fasa, in southern Iran, has an HCV infection rate of 13.3% in hemophilic patients, the lowest in Iran, and Isfahan, in the central region of Iran, has an HCV infection rate of 80.5% among patients with inherited coagulation disorders, the highest in Iran. We found that prevalence of HCV infection among hemophilic patients is significantly lower in southern Iran than in northern and central Iran (Figure 2).

### 4.3. HCV Infection Rate Among Hemophilia Patients in EMRO

**Figure 3:**
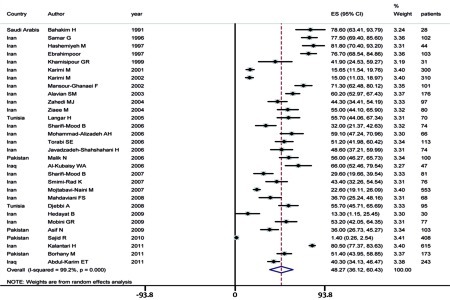
Summary of HCV Infection Rate Estimates in Hemophilic Patients in EMRO Countries

**Figure 4:**
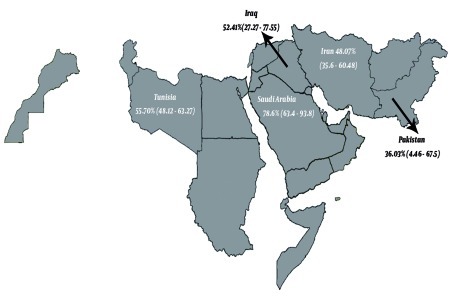
Geographical Distribution of HCV Infection Among Hemophilic Patients in EMRO Countries

The pooled estimate of HCV seroprevalence in hemophilic patients in the EMRO region was 48.27% ([95% CI, range: 36.12–60.43], P value as determined by the Q-squared test ≤ 0.001, I2 value = 99.2%, and Tau-square value = 658.9) (Figure 3). The infection rate was 36.03% ([95% CI, range: 4.46–67.59], P value as determined by the Q-squared test ≤ 0.001, I2 value = 99.2%, and Tau-square value = 1.0e + 03) in Pakistan, and it was estimated as 55.70% ([95% CI: 48.1–63.27] with a P value as determined by the Q-squared test = 1.000, I2 value = 0.0%) for Tunisia by using the fixed-effect approach. The pooled estimate of HCV infection among hemophilic patients in Iraq was 52.41% ([95% CI: 27.27–77.55] P value of Q-squared test was < 0.001, I2 test = 91.3%, and Tau-squared test = 301.4). The infection rate in Saudi Arabia is reported to be 78.6 % (95% CI: 63.4–93.8).Figure 4 shows the geographical distribution of individual or pooled estimates of the HCV infection rate among hemophilic patients in EMRO countries.It seems that genotype 1a is the most common genotype among hemophilic patients; however, because of few reports from the different provinces of EMRO region, it was not possible to consider a pooled estimate of the prevalence of HCV infections. Most studies did not evaluate the risk factors, but some have reported male sex, hemophilia A comparing with B, and the long duration of blood transfusion as risk factors although the odds ratio (OR) was not calculable because of the lack of sufficient number of studies.

## 5. Discussion

Hepatitis C is a major health problem worldwide, and it leads to liver failure, other morbidities, and mortality among infected patients. Patients with inherited coagulation disorders who have received multiple transfusions are at high risk for hepatitis C infection, because HCV-infected blood or blood products are considered the primary sources of HCV transmission [6]. Hemophilia is most common in Qatar, affecting 15.8 patients of each 100,000 male population, but it is a neglected issue in neighboring countries, thus making it an important healthcare burden in these countries. The quality of life of hemophilic patients is similar to that of the general population in developed countries because of safety-factor concentrates, blood transfusion, and a multidisciplinary comprehensive care plan. However, in developing countries, because of the lack of safe blood products, treatment strategies, and registration centers for arranging necessary equipment, and possibly because of cost-related problems, patients are at the risk of acquiring a disability. In addition, according to an estimation in a study, only 25% of all hemophilic patients receive adequate treatment, and unfortunately most of them die before they reach the age of 20 years [42]. Nevertheless, some developing countries in EMRO, like Iran, are making efforts to ensure better care for patients [63]. The incidence of acute hepatitis C in hemophilic patients has reduced because of blood-product screening since the late 1980s and has reached a plateau in recent years [64]. Although the situation of HCV infections in developing countries seems ambiguous, our findings indicate that HCV transmission by blood or blood products has been controlled successfully in Iran and perhaps in other developing countries in EMRO. The HCV infection rate has reached from 81.8% to 13.3% between 1997 and 2009 in Iran. Although laboratory assessment techniques have been developed and these are more sensitive than the techniques used 13 years ago, the reason behind the increase in infection rate in 2011 is not clear. One of the reasons for this increase could be overestimation of values. This is a serious concern for health-policy makers, and they are responsible for identifying the reason for this augmentation and resolve any possible problems. We found that HCV prevalence is significantly lower in southern Iran than in other parts of the country. Although the Sistan-Baluchestan province in southern Iran has a high rate of HCV infection (0.449%) in general population, in the northern provinces of Golestan and Guilan, this infection is prevalent in more than 1% of the general population [65]. In addition, most of the immigrants to Tehran (capital of Iran) are from northern Iran, which may have led to an increase in the HCV-infection rate in central Iran. This reduction in HCV-infection rate can be conservatively considered as true for other EMRO countries as well; however, because of lack of sufficient data from most of these countries, this conclusion should be drawn with caution. HCV prevalence among hemophilic patients in Iran is higher than that in Pakistan. In contrast, the prevalence of hepatitis C among thalassemia patients in Iran is lower than that in any other EMRO country [66]. We think that this difference indicates other sources of transmission in addition to blood transfusion, such as non-screened HCV-infected clotting factor concentrates and unsafe imported blood products. Contradictory reports from Pakistan indicate a discrepancy in the correct evaluation of transmission reduction. Two recent (2010 and 2011) studies from Pakistan have reported very different rates of HCV infection among patients with bleeding disorder (1.4% versus 51.4%); this contradiction may be because of the dissimilarities between them in terms, bias in patient selection, type of transfused products, brand of blood-screening products, and laboratory assessment method. HCV screening is another issue overlooked in hemophilic patients. We found that most of the EMRO countries did not report the HCV prevalence among patients with inherited coagulation disorders, whereas early diagnosis and treatment of these patients is required, because the response to treatment reduces with age [9]. Moreover, the most common genotype among hemophilic patients is genotype 1 [67]; treatment of patients with this genotype is considered difficult and requiring a longer duration than treatment of patients with other genotypes. Therefore, early diagnosis and treatment are really important in hemophilic patients [64].

We found that there is a huge lack of knowledge about patients with inherited coagulation disorders and the complications that they can encounter because of being at high risk for other conditions. Probably, other fields of medicine also face problems arising from such lack of knowledge. Other studies may confirm our hypothesis about developing countries in EMRO. Further studies are warranted for determining similar trends for other diseases and identifying knowledge gaps for some forgotten diseases.

## 6. Conclusion

The HCV infection rate among patients with inherited coagulation disorders is high in Iran and other EMRO countries. It seems that the incidence of transmission of HCV infection through blood products has reduced in Iran. There is a lack of adequate data on hemophilic patients and the complications they may encounter in most of the EMRO countries. It is the responsibility of health-policy makers to address this knowledge gap and provide safe and adequate treatment for patients in high-risk groups.
